# Novel Pyranopyrazoles: Synthesis and Theoretical Studies

**DOI:** 10.3390/molecules170910377

**Published:** 2012-08-30

**Authors:** Ahmed A. Al-Amiery, Redah I. Al-Bayati, Fouad M. Saed, Wassan B. Ali, Abdul Amir H. Kadhum, Abu Bakar Mohamad

**Affiliations:** 1 Applied Chemistry Division, Applied Science Department, University of Technology (UOT), Baghdad 10001, Iraq; 2 Department of Chemical & Process Engineering, University of Kebangsaan Malaysia (UKM), Bangi, Selangor 43000, Malaysia; 3 Chemistry Department, College of Science Department, Al-Mustansirya University, Baghdad 10001, Iraq

**Keywords:** atomic charges, DFT, ethylenediamine, LUMO, pyranopyrazoles

## Abstract

A series of pyranopyrazoles, namely, 7-(2-aminoethyl)-3,4-dimethyl-1-phenyl-1*H*-pyrazolo[3,4-b]pyridin-6(7*H*)-one (**2**), (*Z*)-3,4-dimethyl-1-(4-((4-nitrobenzylidene)amino)phenyl)pyrano[2,3-c]pyrazol-6(1*H*)-one (**5**), 1-(4-(3,4-dimethyl-6-oxopyrano[2,3-c]pyrazol-1(6*H*)-yl)phenyl)-3-(naphthalen-1-yl)urea (**6**), (*Z*)-ethyl 4-((3,4-dimethyl-6-oxo-1,6-dihydropyrano[2,3-c]pyrazol-5-yl)diazenyl)benzoate (**8**) and 3,4-dimethyl-*N*-(naphthalen-1-yl)-6-oxopyrano[2,3-c]pyrazole-1(6H)-carboxamide (**9**) were synthesized and characterized by means of their UV-VIS, FT-IR, ^1^H-NMR and ^13^C-NMR spectral data. Density Functional Theory calculations of the synthesized pyranopyrazoles were performed using molecular structures with optimized geometries. Molecular orbital calculations have provided detail description of the orbitals, including spatial characteristics, nodal patterns, and the contributions of individual atoms.

## 1. Introduction

**Scheme 1:**
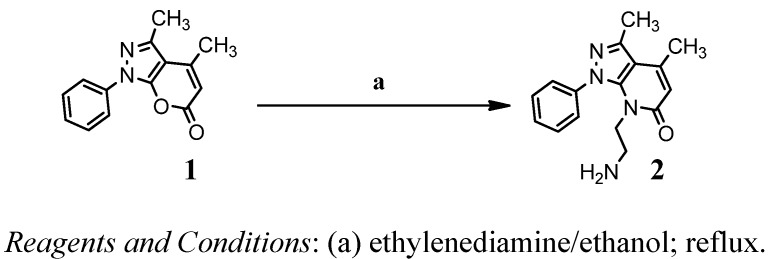
Reaction sequences of synthesis for compound **2**.

Pyranopyrazoles are reported to have useful properties as therapeutics in clinical application [1,2,3]. A literature survey revealed that pyrazole derivatives have received much attention during the recent years on account of their prominent utilization as analgesic, anti-inflammatory, ulcerogenic [4], antibacterial, antifungal [5,6], antitubercular [7], antimalarial [8], antitumor [5,9,10], antioxidant [10], antiproliferative [11], antihypertensive [12], hypnotic [13] and vasodilator [14]. Recently, some pyrazole carbohydrazide derivatives were reported to have moderate anticancer activity [15]. The synthesis of pyrazole derivatives with sugar moieties has also received considerable attention due to their broad spectrum of biological activities [16,17,18,19,20,21,22,23,24,25]. However, a search of the literature revealed that pyrazole-5-carbohydrazide derivatives with a carbohydrate moiety have not been well described; therefore their synthesis and biological study seemed to be an attractive task [26]. In continuation of previous work [27,28,29,30,31,32,33,34,35,36,37,38], we have focused on the synthesis of new heterocyclic compounds, and herein we are reporting the synthesis of new pyranopyrazoles **2**–**9** (Scheme 1–Scheme 3).

**Scheme 2:**
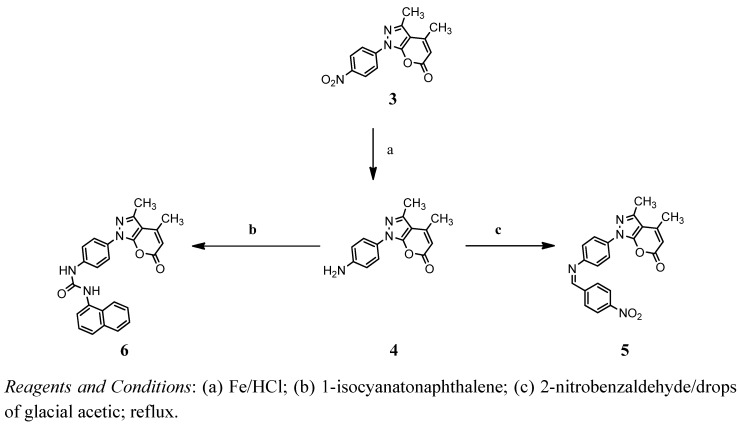
Reaction sequences of synthesis of compounds **5** and **6**.

**Scheme 3:**
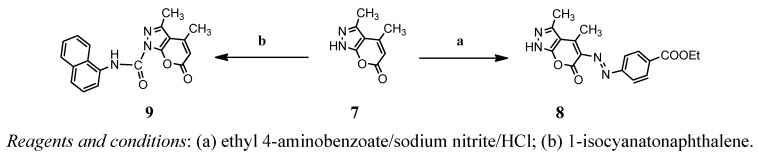
Reaction sequences of synthesis of compounds **8** and **9**.

## 2. Results and Discussion

### 2.1. Chemistry

**Scheme 4:**
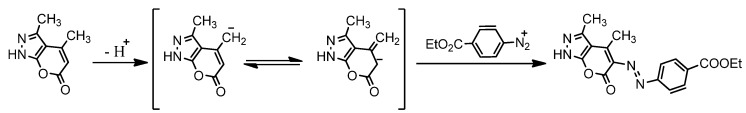
Reaction mechanism of the synthesis of compound **8**.

The synthesis of 1-(4-aminophenyl)3,4-dimethylpyrano[2,3-C]pyrazol-6(1*H*)-one (**4**) was conducted using iron powder and concentrated hydrochloric acid in ethanol. Compounds **5** and **6** were obtained from compound **4** with 2-nitrobenzalaldehyde (in the presence of glacial acetic acid) or 1-isocyanatonaphthalene (Scheme 2). Compound **8** was synthesized by the reaction of compound **7** with 4-(ethoxycarbonyl)benzenediazonium chloride. The reaction of compound **8** with diazonium salt could be visualized to ocurr through the mechanism shown in Scheme 4.

**Scheme 5:**
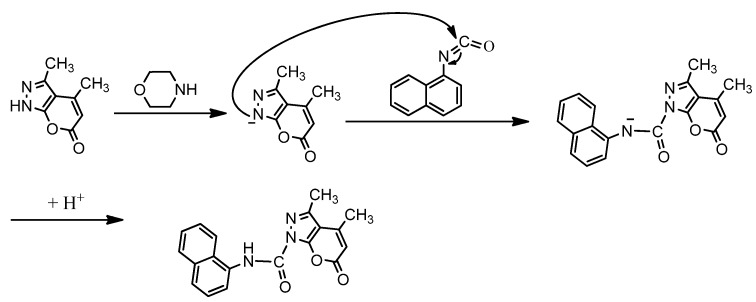
Reaction mechanism of the synthesis of compound **9**.

Compound **9** was synthesized via the reaction of compound **7** with 1-isocyanatonaphthalene in the presence of morpholine. The formation of compound **9** could be visualized to ocurr through the mechanism shown in Scheme 5.

### 2.2. Compound Characterizations

The first three pyranopyrazole compounds were prepared in this work and these compounds were used as the starting material for further syntheses (Scheme 1–Scheme 3). Compound **1** was reacted with ethylenediamine in ethanol as a solvent to obtain compound **2**. The structure of the synthesized compound has been characterized and identified by UV, FTIR and NMR spectrum. The FTIR shows an absorption band at 3294 cm^−1^ due to the NH_2_ stretching vibration and a band at 1,683 cm^−1^ for amide C=O, which appeared at 1755 cm^−1^ in the pyranopyrazole-6-one compound **1**. Through the ^1^H-NMR spectrum of compound **2** the following data were obtained: 6.7–7.9 (6H, Ar-H), 6.0 (s, 1H, Ar-H), 3.1 (m, 2H, CH_2_NH_2_), 3.5 (t, CH_2_CH_2_), 2.57 (s, 6H, CH_3_), 2.2 (t, 2H, NH_2_). The ^13^C-NMR spectrum of compound **2** shows the following bands for carbon: 12, 24, 42, 47, 90, 118, 120–129, 145, 151, 170. Schiff’s base (**5**) has been synthesized by condensation of compound **4** with 2-nitrobenzaldehyde in absolute ethanol as a solvent with an addition of few drops of glacial acetic acid. Compound **5** shows an absorption band at 1630 cm^−1^ due to stretching vibration of C=N moiety. The ^1^H-NMR spectrum of compound **5** shows the absorption peaks to be as follows: 9.1 (s, 1H, for -N=CH), 7.3–8.2 (8H, Ar-H), 6.6 (s, 1H, Ar-H), 2.5 (s, 6H, CH_3_). Compound **6** has been prepared by treatment of compound **4 **with an equimolar quantity of 1-isocyanatonaphthalene. The FTIR spectrum of compound **6** showed the disappearance of the absorption band for NH_2_ and the appearance of new band due to NH. The ¹H-NMR spectrum of compound **6** shows the following data: 8.1–8.7 (4H, Ar-H), 6.3 (s, 2H, N-H), 5.7 (s, 1H, Ar-H), 2.68 (s, 6H, CH_3_). The ^13^C-NMR spectrum of compound **6** shows the following carbon peaks: 15, 21, 109, 116, 128, 120–129, 147, 148, 158 and 162 ppm. Compound **8** has been prepared by treatment of compound **7** with an equimolar quantity of 1-isocyanatonaphthalene (Scheme 4). The FTIR spectrum of compound **9** showed the appearance absorption bands of NH moiety at 3269 cm^−1^ with another absorption band at 1701 cm^−1^ due to the carbonyl group moiety of lactone and the appearance of a new absorption band of C=O for amide moiety at 1633 cm^−1^. The ^1^H-NMR spectrum of compound **9** shows the following data at 6.09 (s, 1H, Ar-H), 2.2 (m, 2H, CH_2_CH_3_), 2.7 (s, 3H, CH_3_), 2.6 (s, 3H, CH_3_), 4.4 (m, 1H, NHCH_2_) 1.2 (t, 3H, CH_2_CH_3_). The last compound **8** was prepared by reaction with compound **7** with the diazonium salt of ethyl 4-aminobenzoate. The FT-IR spectrum of compound **8** showed the appearance of the characteristic absorption band at 1672 cm^−1^ due to the stretching vibration of the carbonyl group moiety of ester, while the absorption band at 1705 cm^−1^ was due to the stretching vibration of the C=O lactone moiety, 1606 cm^−1^ for C=N moiety and the frequency at 1562 cm^−1^ was due to N=N stretching vibration. The ^13^C-NMR spectrum of compound **8** shows the following peaks: 12, 14, 61, 115, 125–131, 145, 160, 165.

### 2.3. Computational Studies

#### 2.3.1. Atomic Charges

**Figure 1:**
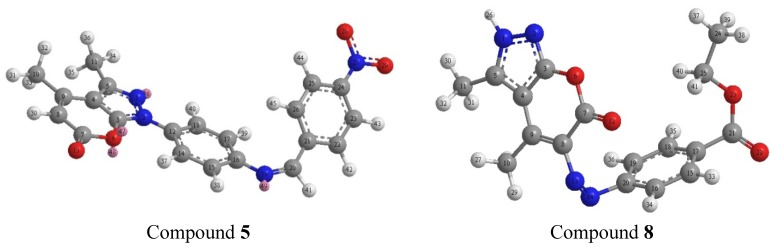
Optimized 3D geometrical structures for compounds **5** and **8**.

An earlier study [27,28,30,32,33] had shown that the atomic charges were affected by the presence of the substituent of the rings. With the aid of a reference model, compounds **5** and **8** with optimized geometries and 3D geometrical structures are given in Figure 1. The data showed that the highest atomic charge in compound 5 is at [O(29)-0.998012] followed by the next charge value at [O(28)-0.991082; O(13)-0.639886 and N(1)-0.394836]. These data clearly showed that these atoms are most reactive toward the addition substitution reactions. The determined bond angle and twist angle, stretch (2.2196), bend (22.6468), stretch-bend (0.0068), torsion (−6.5971) and the 3D geometrical structure indicated that this molecule is a non-planar molecule with the stereochemistry at [C(9)-C(8): (Z) and N(19)-C(20): (Z)]. The highest atomic charge in compound **8** is at [O(22)-0.895221] followed by the next charge value at [O(12)-0.766002; N(2)-0.357642 and C(19)-0.335865]. These data indicated that these atoms are most reactive toward the addition substitution reactions. The determined bond angle and twist angle, stretch (6.3552), bend (96.6357), stretch-bend (−0.3675), torsion (11.2595) and the 3D geometrical structure indicated that this molecule is also a non-planar molecule with [C(9)-C(8): (E) and N(13)-N(14): (Z)] as the stereochemistry.

#### 2.3.2. Density Function Theory (DFT)

**Figure 2:**
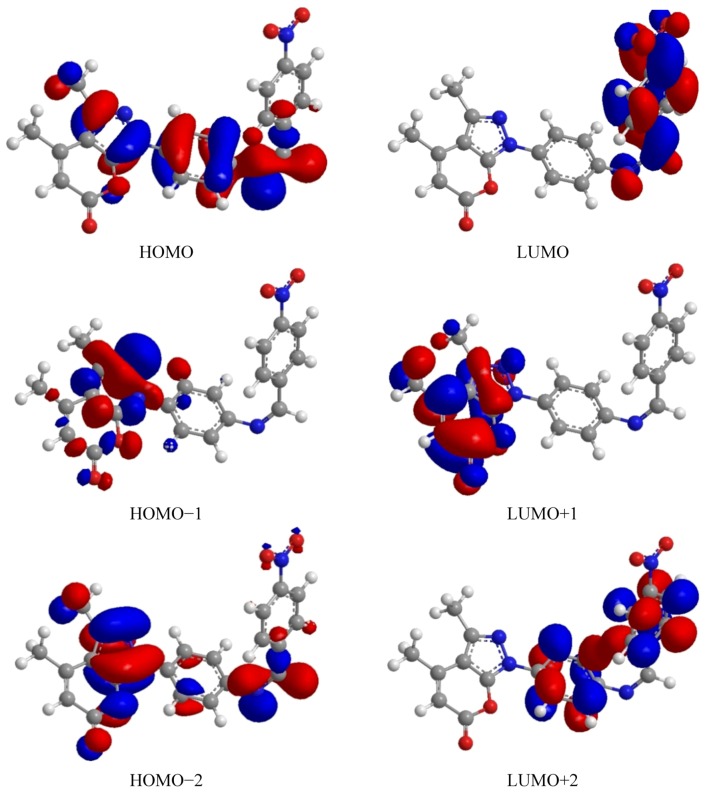
Highest occupied molecular orbital (HOMO) and the lowest unoccupied molecular orbital of compound **5**.

**Figure 3:**
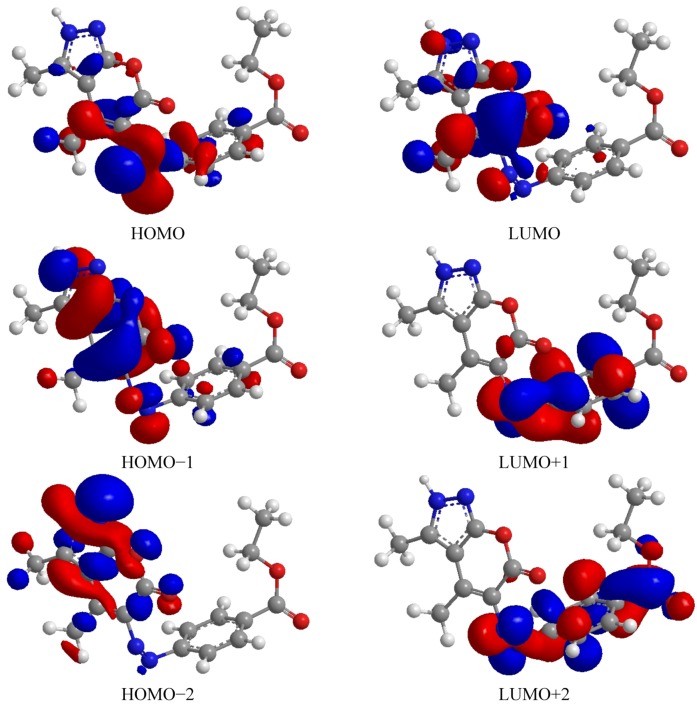
Highest Occupied molecular orbital and the lowest unoccupied molecular orbital of compound **8**.

DFT calculations have been performed for compounds **5** and **8**. The optimized molecular structure of the most stable form is shown in Figure 1 The calculated energies are presented in Table 1. Molecular orbital calculations could provide a detailed description of the orbitals including the spatial characteristics, nodal patterns and individual atom contributions. The contour plots of the frontier orbitals for the ground state of compounds **5** and **8** are shown in Figure 2 and Figure 3 together with the Highest Occupied Molecular Orbital (HOMO) and the Lowest Unoccupied Molecular Orbital (LUMO). It is interesting to observe that both orbitals are substantially distributed over the conjugation plane. In addition, it can also be seen from Figure 2 and Figure 3 that the HOMO orbitals are located on the substituted molecule while LUMO orbitals resemble those obtained for the unsubstituted molecule and therefore the substitution has contributed an influence on the electron donation ability while imposing only a small impact on electron acceptance ability. The orbital energy levels of HOMO and LUMO of compounds **5** and **6** are listed in Table 2. An electronic system with a larger HOMO-LUMO gap should be less reactive compare to the one having a smaller gap. In the present study, the HOMO-LUMO gap values of compound **5** are at −4.65, −8.255 and −10.226 eV while for compound **8** are situated at −2.666, −9.345 and −10.398 eV. It can be seen that the energy gaps between HOMO and LUMO is −4.65 eV for compound **5**, and −2.666 eV for compound **8**. The lower value in the HOMO and LUMO energy gap would explain the eventual charge transfer interaction taking place within the molecules. The low values of HOMO for compounds **5** and **8** indicate that these molecules have low ionization energies inferring that they can lose the electrons easily thus indicating that compounds **5** and **8** are potentially good corrosion inhibitors [39]. The dipole moments of compounds **5** and **8** were also calculated and listed in Table 1.

**Table 1:** Total energy and dipole moments (Debye) for compounds **5** and **8**.

Compound	Total Energy	Dipole Moments
**5**	56.0882 kcal/mol	7.1113
**8**	167.4106 kcal/mol	15.3621

**Table 2:** HOMO and LUMO energies (eV) of compounds **5** and **8**.

Comp.	HOMO	LUMO	∆E	HOMO−1	LUMO+1	∆E	HOMO−2	LUMO+2	∆E
**5**	−9.383	−4.732	−4.651	−10.833	−2.578	−8.255	−11.552	−1.326	−10.226
**8**	−4.715	−2.094	−2.666	−10.597	−1.252	−9.345	−11.314	−0.917	−10.398

The UV-VIS absorption spectrum of compound **5** was recorded in the ethanol solution. The absorption peaks are observed at 325 and 202 nm and 329 and 267 nm for compounds **5** and **8**, respectively. It can be deduced that these peaks imply to the n→p* and p→p* transitions. The 3D plots of the HOMO−2, HOMO−1, HOMO, LUMO, LUMO+1, LUMO+2 and the corresponding energy levels for compounds **5** and **8** are shown in Figure 2 and Figure 3, respectively. The theoretical electronic transfers (ET) for compound **5** were at 336, 217 nm and for compound **8** were at 341 and 233 nm corresponding to the UV-VIS spectral absorption peaks and the electronic transfers of HOMO plus LUMO and HOMO plus LUMO+1, respectively. The increased theoretical absorption wavelengths of the compounds have slight blue-shifts when compared to the corresponding experimental absorption wavelengths.

Figure 2 and Figure 3 shows the six main orbitals that have contributed in the vertical electronic transitions for compounds **5** and **8**. These orbitals, namely, HOMO−2, HOMO−1, HOMO, LUMO, LUMO+1 and LUMO+2, represent the three highest occupied orbitals and three lowest unoccupied orbitals in compounds **5** and **8**. Similar spatial distribution of orbitals between HOMO/HOMO−1/HOMO−2 and LUMO/LUMO+1/LUMO+2 pairs and the population analysis for compound **5** indicates that the electronic transitions and the electron clouds of the HOMO are delocalized on the *N*-*N*-pryazole bridge, benzene ring and also on the *N*-benzyl bridge with benzene ring. However, HOMO−1 is delocalized on *N*-*N*-pyrazole bridge, while the HOMO−2 is delocalized on *N*-*N*-pyrazole bridge and also *N*-benzyl bridge. These orbitals are of the p-type bonding orbital. LUMO is found mainly delocalized on the nitro group while LUMO+1 is delocalized on the pyrazole ring. For the LUMO+2 it is mainly delocalized on benzyl ring and *N*-*N*-pyrazole bridge. For compound **8**, the population analysis indicates that the electronic transitions and the electron clouds of the HOMO are delocalized on the N=N-bridge with the HOMO-1 and HOMO-2 being delocalized on the pryrazole having the p-type bonding orbital. LUMO is found mainly delocalized on the pyrazole ring while LUMO+1 and LUMO+2 are delocalized on the N=N-bridge. In all cases, the LUMOs exhibited p*-type anti-bonding orbitals.

## 3. Experimental

### 3.1. General

The chemicals used during synthesis were supplied by Sigma-Aldrich while the purity of the compounds was checked on the thin layer chromatography (TLC) plates (Silica gel G). The FTIR spectra was obtained on a Shimadzu FTIR-3800 spectrometer on KBr disk. window. The UV-VIS spectra were measured in ethanol using a Shimadzu UV-VIS Model 160A spectrophotometer in the range 200–1,000 nm. The NMR spectra was obtained on Bruker 300 MHz spectrophotometer. A Gallenkamp M.F.B.600.010 F melting point apparatus was used to measure the melting points of all the prepared compounds. 

### 3.2. Synthesis of Compounds ***1***, ***3*** and ***7***

The method of synthesis was from ethylacetoacetate and hydrazine derivatives according to reference [40].

*3,4-dimethyl-1-phenylpyrano[2,3-c]pyrazol-6(1H)-one* (**1**): MP. = 140–142 °C, yield = 85%, and recrystallization solvent is ethanol.

*3,4-dimethyl-1-(4-nitrophenyl)pyrano[2,3-c]pyrazol-6(1H)-one* (**3**): MP. = 243–245 °C, yield = 90%, and recrystallization solvent is ethanol.

*3,4-dimethylpyrano[2,3-c]pyrazol-6(1H)-one* (**7**): MP. = 180–182 °C, yield = 81%, and recrystallization solvent is ethanol.

### 3.3. Synthesis of Compounds ***2***, ***4***, ***5***, ***6***, ***8*** and ***9***

*7-(2-Aminoethyl)-1,3,4-trimethyl-1,7-dihydro-6H-pyrazolo[3,4-b]pyridin-6-one* (**2**)*.* Compound **1** (0.02 mol) was refluxed with ethylenediamine (0.2 mol) in absolute ethanol (30 mL) for 24 hours. The solvent was concentrated and the separated solid product was filtered, dried and recrystallized from ethanol. M.P. = 110–112 °C, yield = 60%. λ_max_ = 221 and 300 nm in ethanol. FTIR: 3429 cm^−^^1^ (N-H stretching vibrations, NH_2_); 3088 cm^−^^1^ (C-H_ar_); 2982 cm^−^^1^ (CH_al_); 1683 cm^−^^1^ (C=O); 1519 cm^−^^1^ (C=C) and 1610 cm^−^^1^ (C=N). ^1^H-NMR (DMSO-*d_6_*): 6.7–7.9 (6H, Ar-H), 6 (s, 1H, Ar-H), 3.1 (m, 2H, CH_2_NH_2_), 3.5 (t, CH_2_CH_2_), 2.57 (s, 6H, CH_3_), 2.2 (t, 2H, NH_2_). ^13^C-NMR (DMSO-*d_6_*): 12, 24, 42, 47, 90, 118, 120–129, 145, 151, 170 ppm.

*1-(4-Aminophenyl)3,4-dimethyl pyrano[2,3-C]pyrazol-6(1H)-one* (**4**). Iron powder (4.0 g) was added portionwise to a mixture of compound **3** (0.01 mol), concentrated hydrochloric acid (15 mL) and ethanol (10 mL). The reaction mixture was refluxed for 6 hours, cooled and the precipitate formed was filtered off, washed with water, dried and recrystallized from ethanol. M.P. = 300 °C, yield =70%. λ_max_ = 206 and 334 nm in ethanol. FTIR: 3250 cm^−1^ (N-H stretching frequencies, NH_2_); 3078 cm^−1^ (C-H_al_); 2968 and 2968 cm^−1^ (C-H_ar_); 1735 cm^−1^ (C=O); 1575 cm^−1^ (C=C) and 1606 cm^−1^ (C=N).

*(Z)-3,4-Dimethyl-1-(4-((4-nitrobenzylidene)amino)phenyl)pyrano[2,3-c]pyrazol-6(1H)-one* (**5**). To a solution of compound **4** (0.001 mol) in absolute ethanol (20 mL), 2-nitrobenzalaldehyde (0.001 mol) was added with 3–4 drops of glacial acetic acid. The mixture was refluxed for 6 hours, cooled then the solid formed was filtered and recrystallized from ethanol. M.P. = 246–248 °C, yield = 80%. λ_max_ = 202 and 325 nm in ethanol. FTIR: 3067 cm^−^^1^ (C-H_ar_); 2899 cm^−^^1^ (C-H_al_); 1740 cm^−^^1^ (C=O); 1560 cm^−^^1^ (C=C) and 1630 cm^−^^1^ (C=N). ^1^H-NMR (DMSO-*d_6_*): 9.1 (s, 1H, N=CH), 7.3–8.2 (8H, Ar-H), 6.6 (s, 1H, Ar-H), 2.5 (s, 6H, CH_3_).

*1-(4-(3,4-Dimethyl-6-oxopyrano[2,3-c]pyrazol-1(6H)-yl)phenyl)-3-(naphthalen-1-yl)urea* (**6**). A mixture of compound **4** (0.002 mol) and 1-isocyanatonaphthalene (0.002 mol) in absolute ethanol (25 mL) was refluxed for 8 hours. The solvent is then concentrated, cooled and the separated solid product was filtered and recrystallized from benzene. M.P. = 162–164 °C, yield = 60%. λ_max_ = 222 and 338 nm in ethanol. FTIR: 3280 and 3170 cm^−^^1^ (N-H stretching frequencies); 3080 cm^−^^1^ (C-Har); 2933 and 2985 cm^−^^1^ (CHal); 1720 and 1670 cm^−^^1^ (C=O); 1560 cm^−^^1^ (C=C) and 1597 cm^−^^1^ (C=N). ^1^H-NMR (DMSO-*d_6_*): 8.1–8.7 (14H, Ar-H), 6.3(s, 2H, N-H), 5.7 (s, 1H, Ar-H), 2.68 (s, 6H, CH_3_). The 13C-NMR: 15, 21, 109, 116, 128, 120–129, 147, 148, 158, 162.

*(Z)-3,4-Dimethyl-5-(phenyldiazenyl)pyrano[2,3-c]pyrazol-6(1H)-one* (**8**). Ethyl 4-aminobenzoate (0.01 mol) is added to a solution of water (4 mL) and concentrated hydrochloric acid (2.25 mL). The resulting solution is stirred for 10 minutes before being cooled to 0–5 °C. A solution of sodium nitrite (0.011 mol, 0.76 g) in water (2.5 mL) is added dropwise. After being stirred for 10 minutes, resulting solution of diazonium salt was added dropwise to a mixture of compound **7** (0.012 mol) in ethanol and 10% NaOH (10 mL) at 0 °C to 5 °C and pH = 5.5. After the addition is completed, the mixture was stirred for further 20 minutes and then was left for 1 hour. The resulting solid compound **8** was filtered, washed with water, dried and recrystallized from ethanol. M.P. = 158–160 °C, yield = 73%. λ_max_ = 267 and 329 nm in ethanol. FTIR: 3296 cm^−^^1^ (N-H stretching frequeny); 3070 cm^−^^1^ (C-Har); 2935 and 2982 cm^−^^1^ (C-Hal); 1705 and 1672 cm^−^^1^ (C=O); 1519 cm^−^^1^ (C=C) and 1606 cm^−^^1^ (C=N). ^13^C-NMR (DMSO-*d_6_*): 12, 14, 61, 115, 125–131, 145, 160, 165 ppm.

*3,4-dimethyl-N-(naphthalen-1-yl)-6-oxopyrano[2,3-c]pyrazole-1(6H)-carboxamide* (**9**). A mixture of compound **7** (0.01 mol) and 1-isocyanatonaphthalene (0.01 mol) was dissolved in DMF (20 mL) in a round bottom flask and two to three drops of morpholine were added as a catalyst. Then the well-stirred mixture was refluxed for 6–8 hours and then allowed to stand at room temperature before pouring into ice-cold water. The resulting solid product was filtered, dried, and recrystallized from dioxane. M.P. = 171–173 °C; yield = 86%; λ_max_ = 220 and 344 nm in dioxane. FTIR: 3269 cm^−^^1^ (N-H stretching frequency); 3053 cm^−^^1^ (C-H_ar_); 2926 and 2860 cm^−^^1^ (C-H_al_); 1700 and 1633 cm^−^^1^ (C=O); 1610 cm^−^^1^ (C=C). ¹H-NMR (DMSO-*d_6_*): 6.09 (s, 1H, Ar-H), 2.2 (m, 2H, CH_2_CH_3_), 2.7 (s, 3H, CH_3_), 2.6 (s, 3H, CH_3_), 4.4 (m, 1H, NHCH_2_), 1.2 (t, 3H, CH_2_CH_3_).

### 3.4. DFT

The molecular representation sketch of the reference compound was plotted using ChemBioOffice 2010 software. All the quantum chemical calculations were performed using the Density Functional Theory (DFT) methodology with 3–21G basis set, while the molecular atomic charges were calculated via the Mulliken population analysis.

## 4. Conclusions

In this study, the compounds labeled **1**–**9** have been synthesized and characterized using various spectroscopic methods and elemental analysis technique. The synthesized compounds **5** and **8** were studied theoretically and their atomic charges and stereochemistry were estimated and it was found that they are not planar. 
